# FTO Facilitates Cervical Cancer Malignancy Through Inducing m6A‐Demethylation of PIK3R3 mRNA

**DOI:** 10.1002/cam4.70507

**Published:** 2024-12-18

**Authors:** Bingxin Chen, Liming Wang, Xiaomin Li, Ci Ren, Chun Gao, Wencheng Ding, Hui Wang

**Affiliations:** ^1^ Department of Obstetrics and Gynecology, Tongji Hospital, Tongji Medical College Huazhong University of Science and Technology Wuhan China; ^2^ Department of Gynecologic Oncology, Women's Hospital Zhejiang University School of Medicine Hangzhou China

**Keywords:** cervical carcinoma, FTO, m6A, malignancy, PIK3R3

## Abstract

**Background:**

The incidence rate and mortality of cervical cancer rank the fourth in the global female cancer. N6‐methyladenosine (m6A) always plays an important role in tumor progression, and fat mass and obesity‐associated gene (FTO) works as the m6A demethylase.

**Aims:**

Our study aimed to narrate the biological function and potential mechanisms for FTO in cervical cancer malignancy.

**Materials & Methods:**

We analyzed potential clinical value of FTO in cervical cancer patients. The relative protein levels of FTO in cervical cancerous tissue and paracancerous tissue were verified by IHC. After changing the FTO expression level by lentivirus transfection, the proliferation and metastasis ability of cervical cancer cells were detected both in vitro and in vivo. Further, Merip‐seq and Merip‐qPCR are used to profile m6A transcriptome‐wide. Finally, western blot were performed to identify the regulatory mechanism.

**Results:**

Based on TCGA‐CESC cohort and GEO dataset, FTO expression levels in HPV‐positive cancer patients were significantly higher than those in HPV‐negative cancer patients and could predict advanced FIGO stage. The protein level of FTO in cervical cancerous tissue was higher than that in paracancerous tissue. Functional assays indicated that FTO promoted the proliferation, migration and invasion of cervical cancer cells both in vitro and in vivo. The Merip‐seq and Merip‐qPCR evoked that relative PIK3R3 m6A level was significantly increased after FTO knockdown, which effected the activation of FoxO pathway. After knocking down FTO, upregulation of PIK3R3 can restore the malignancy of cervical cancer.

**Conclusion:**

All in all, these data suggest a vital role for FTO in occurrence and development of cervical cancer.

## Graphical abstract

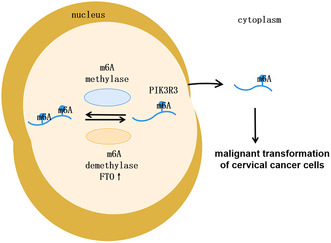

## 1 Introduction

The incidence rate and mortality of cervical cancer rank the fourth in the global female cancer [1]. The 5‐year survival rate of patients with advanced cervical cancer is less than 20% [2, 3, 4]. The main factors affecting the survival are inevitable recurrence and metastasis [5, 6, 7]. The growing evidence displayed that the cervical cancer progression was a complex process, which involved the changes in the levels of genetics, epigenetics and transcriptomics [8, 9]. Therefore, the factors associated with cervical cancer malignancy are the hot candidates in cervical cancer, which might provide new ideas for early diagnosis and targeted therapy.

Epigenetic regulation plays an important role in the biological behavior of malignant tumor. N6‐methyladenosine (m6A) modification is an important epigenetic regulations, and work as a crucial regulator for gene expression at the post‐transcriptional level [10, 11, 12, 13]. There are three major kinds of enzymes involved in m6A modification: Writers, Erasers, and Readers. The sixth nitrogen atom of RNA adenine base is methylated through Writers, which mainly include Methyltransferase like 3 (METTL3), Methyltransferase like 14 (METL14) and Wilms tumor 1‐associating protein (WTAP) [14, 15, 16]. Eraser, mainly including fat mass and obesity‐associated gene (FTO) and ALKB homolog 5 (ALKBH5), can demethylate the m6A‐modified RNA [17, 18]. Writers and Erasers cooperate to complete the reversible m6A modification, and the m6A‐modified RNA is recognized by specific Readers, mainly including YT521‐B homology (YTH) domain family proteins (YTHDF1/2/3) and YTH domain containing proteins (YTHDC1/2) [19, 20, 21, 22]. The recognition by Readers determines the splicing, translation and stability of RNA. Therefore, abnormal m6A modification can affect the key gene expression, and play an important role in malignancy of various cancers [23, 24, 25, 26].

Abnormal activation of Forkhead box class O (FoxO) pathway is considered as an important malignant tumor promoter [27, 28, 29]. Class I Phosphoinositide 3‐Kinase (PI3K) is composed of a catalytic subunit‐p110 and a regulatory subunit‐p85, and can trigger the phosphorylation cascade in FoxO pathway [30, 31]. In addition, p55γ works as one of three isoforms in the p85, which is encoded by Phosphoinositide‐3‐kinase regulatory subunit 3 (PIK3R3) [30]. The carcinogenic role of PIK3R3 has been confirmed in several malignant cancers [32, 33, 34, 35, 36, 37, 38].

However, it is unclear whether there is a relation exists between m6A modification and FoxO pathway activity in cervical cancer, as well as their roles in cervical cancer malignancy. In this study, we aimed to identify the novel role of FTO‐dependent m6A modification in cervical cancer malignancy, as well as the profiled m6A transcriptome‐wide mapping. The results evoked that upregulation of FTO promoted the translation of PIK3R3, which enhanced the activation of FoxO pathway and facilitates cervical cancer malignancy.

## 2 Materials and Methods

### 2.1 Cervical Cancer Tissue Sections and Cell Lines

We purchased tissue microarray of cervical cancer patients for immunohistochemistry (IHC), and the clinical pathological characters of included patients was shown in Table S1. The staining results were analyzed through Image J software [39]. Four human cervical cancer cell lines, including C33A (HPV negative), HeLa (HPV 18 positive), SiHa (HPV 16 positive) and CaSki (HPV 16 positive), were purchased from the American Type Culture Collection (ATCC). The human cervical keratinocytes' cell line, S12 (HPV 16 positive), was a gift from Professor Kenneth Raj (Health Protection Agency), with the permission of the original owner, Professor Margaret Stanley. C33A, Hela, and SiHa cells were cultured in Dulbecco's Modified Eagle's medium (DMEM) containing 10% fetal bovine serum (FBS). CaSki cells were cultured in Roswell Park Memorial Institute 1640 medium containing 10% FBS. S12 cells were cultured in DMEM/Ham's F‐12 medium with 10% FBS, insulin, adenine, hydrocortisone, cholera toxin, and epidermal growth factor. The cell lines were cultured in the 37°C incubator with 5% CO_2_.

### 2.2 Establishment of Cells With FTO Knockdown or Overexpression

Lentivirus constructing of FTO knockdown or overexpression was obtained from Genechem Corp. When the cells were adherent to the wall, lentivirus (titer: 2.5 × 10^8^) were added into medium for 24 h according to the manufacturer's instructions. After selection with puromycin, we established cervical cancer cells with FTO knockdown or overexpression.

For the FTO knockdown lentivirus, the sequence gcAGCATACAACGTAACTTTG was cloned into the hU6‐MCS‐Ubiquitin‐EGFP‐IRES‐puromycin lentivirus. The FTO knockdown cells and control cells were established in SiHa cells, which were named as SiHa‐lv‐shFTO and SiHa‐lv‐shcon, respectively. For the FTO overexpression lentivirus, the sequence was cloned into the Ubi‐MCS‐3FLAG‐SV40‐EGFP‐IRES‐puromycin lentivirus. The FTO overexpression cells and control cells were established in CaSki cells, which were named as CaSki‐lv‐FTO and CaSki‐lv‐con, respectively.

### 2.3 Plasma Transfection

Transfection was performed at 60%–70% confluence. The preparation of transfection solution is as follows: solution A: 200 μL opti‐MEM and 4 μg plasmid; solution B: 200 μL opti‐MEM and 10 μL lipo2000. Solution A and solution B were mixed gently, and stand for 20 min. Finally, the transfection solution was added into serum‐free medium.

### 2.4 RNA Extraction and Rt‐qPCR

Cells were lysed by TRIZOL, and then chloroform, isopropanol, and 75% ethanol were added in turn. The precipitated RNA was dissolved in RNase‐free water and its concentration was measured by NanoDrop. After reverse transcription of RNA into cDNA, Ct values of target genes and GAPDH were detected by the CFX96 TouchTM fluorescent quantitative PCR instrument and performed the RT‐qPCR analysis of RNA.

The primers for FTO:

Forward: 5′‐CCAGAACCTGAGGAGAGAATGG‐3′.

Reverse: 5′‐CGATGTCTGTGAGGTCAAACGG‐3′.

The primers for PIK3R3:

Forward: 5′‐CTGACATTTAATTCCGTGGTGGA‐3′.

Reverse: 5′‐TCAAGTTTGGGATTGTACTGAGC‐3′.

The primers for GAPDH:

Forward: 5′‐CGGATTTGGTCGTATTGGG‐3′.

Reverse: 5′‐CTGGAAGATGGTGATGGGATT‐3′.

### 2.5 Protein Extraction and Western Blot

Protein was isolated using RIPA plus Cocktail and phenylmethylsulfonyl fluoride (PMSF). The concentration of the extracted protein liquid was measured by the BCA protein concentration kit. Samples were separated on 10% sodium dodecyl sulfate‐polyacrylamide gel electrophoresis (SDS‐PAGE) gel and transferred to the activated polyvinylidene fluoride (PVDF) membrane. The following specific primary antibodies were incubated with the membranes at the indicated dilution: anti‐FTO (1:1000, ab126605, abcam), anti‐PIK3R3 (1:1000, 11,889, Cell Signaling Technology), anti‐p‐AKT (ser473) (1:5000, ab81283, abcam), anti‐AKT (1:500, ab8805, abcam), and anti‐GAPDH (1:5000, ab9484, abcam). The membranes were incubated with HRP‐conjugated anti‐mouse IgG (1:10,000, 7076, Cell Signaling Technology) or anti‐rabbit IgG (1:10,000, 7074, Cell Signaling Technology). Finally, the PVDF membrane were detected by the ChemiDocXRS and imaging system.

### 2.6 Measurement of m6A Level

M6A level in total RNA was measured by EpiQuik M6A RNA Methylation Quantification Kit. After adding RNA samples, negative control and positive control to the detection wells, binding solution, capture antibody, detection antibody, enhancer solution, and development solution were incubated in turn. When the color of the positive control turns blue, stop solution was added into detection wells. The m6A levels were quantified colorimetrically by reading the absorbance of each well at a wavelength of 450 nm (OD450) within 2–15 min.

### 2.7 Immunocytochemistry

Dimethylbenzene solution was used for dewaxing, and alcohol with gradient concentration was used for dehydration. Antigenically repair process was performed on tissue sections, and then endogenous peroxidase was blocked. The tissue sections were incubated with the specific primary antibody for anti‐FTO (1:500, ab126605, abcam). The tissue sections were visualized using microscope and scored according to the Image J software.

### 2.8 Cell Counting Kit‐8 (CCK8) Assay

For the assay, 100 μL cell suspension containing 8000 cells was prepared and inoculated in 96 well plates per well. After incubation, 10 μL CCK‐8 solution was added to each well. The absorbance value at 450 nm was measured by microplate reader.

### 2.9 Wound Healing Assay

When the cells in the 6‐well cell culture plates were full, scratches were drawn by pipette tip with the assistance of a ruler. After 0 and 24 h, pictures were taken through microscope.

### 2.10 Transwell Migration and Invasion Assay

For the transwell migration assay, the upper chamber of the transwell plate was added with 100 μl cell suspension without FBS, and the lower chamber was added with 600 μl of complete medium. When the cells in the upper chamber pass through the membrane and join into the lower chamber, the upper chamber was washed with PBS, fixed in 70% ethanol for 10–15 min at room temperature, and stained with 0.2% (w/v) crystal violet and incubate for 3–5 min at room temperature in turn. Also, for the transwell invasion assay, 40 μl of diluted matrigel that mixed with serum‐free medium at a ratio of 1:8 was spread on the bottom membrane of the upper chamber. The other steps were the same as transwell migration assay [40].

### 2.11 Ki67 Assay

Cell suspension were prepared. While vortexing, 3 mL of pre‐chilled 70% ethanol was gradually added to the cell suspension. After being incubated at −20°C for 1 h, cells were stained with Ki‐67 antibody for 30 min at room temperature in the dark. The staining outcomes were examined through flow cytometry for analysis.

### 2.12 Subcutaneous Tumor Model

Eight 4‐week‐old female nude mice were purchased from Gempharmatech Co. Ltd. and were randomly divided into two groups. 3 × 10^7^ SiHa‐lv‐shFTO or SiHa‐lv‐shcon cells were slowly injected near the thigh. Subcutaneous tumor size was measured every 5 days. After feeding for 7 weeks, subcutaneous tumor was dissected and measured. Our animal experiment research was carried out strictly in accordance with the “Guidelines for the Welfare of Experimental Tumor Animals”.

### 2.13 In Vivo Lymph Node Metastasis Model

Eight 4‐week‐old female nude mice were purchased from Gempharmatech Co. Ltd. and were randomly divided into two groups. In order to establish the in vivo lymph node metastasis model, 3 × 10^6^ SiHa‐lv‐shFTO or SiHa‐lv‐shcon cells were slowly injected into the foot pads that containing abundant lymph nodes. After feeding for 20 weeks, we euthanized the nude mice. Lymph nodes in the groin area are dissected and embedded in paraffin for hematoxylin–eosin staining. Our animal experiment research was carried out strictly in accordance with the “Guidelines for the Welfare of Experimental Tumor Animals”.

### 2.14 Methylated RNA Immunoprecipitation Sequencing (Merip‐Seq) and Analysis

Total RNA was extracted from SiHa‐lv‐shFTO and SiHa‐lv‐shcon cells. RNA fragmentation reagents randomly fragmented RNA into 100 nucleotides. According to Magna MeRIP m6A kit, the fragmented RNA was incubated with m6A antibody in IPP buffer for 2 h at 4°C. Then the mixture was immune precipitated for 2 h at 4°C. Methylated RNA was eluted by competition with free m6A, and extracted with RNeasy kit (Qiagen). Purified RNA was used to generate the RNA sequence library using NEBNext Ultra II Directional RNA Library Prep Kit (New England Biolabs Inc., USA). Both the IP samples and the input samples without immunoprecipitation were subjected to 150 bp paired‐end reads through Illumina Hiseq 4000, and were quality controlled by Q30. To analyze Merip‐seq data, we used MetaPlotR to plot the distribution map of m6A methylation peaks on the metagene, and draw the metaGene peak density map. And we took the sequences of the top 2000 peaks with the highest enrichment factor (50 bp on each side of the vertex), and use Dreme to scan the sequences of these peaks to find meaningful motif sequences. Furthermore, Gene Ontology (GO) enrichment analysis and Kyoto Encyclopedia of Genes and Genomes (KEGG) enrichment analysis were conducted in RNA with significant differences in m6A methylation modification.

### 2.15 Merip‐qPCR

First, RNA was divided into 100 nucleotides or smaller fragments, and then they were enriched by immunoprecipitation with monoclonal antibodies against m6A. After immunoprecipitation, the isolated RNA fragments and corresponding input RNA were analyzed by quantitative RT‐PCR.

## 3 Results

### 3.1 FTO Is Overexpressed in Cervical Cancer and Predicts the Late FIGO Stage

**FIGURE 1:**
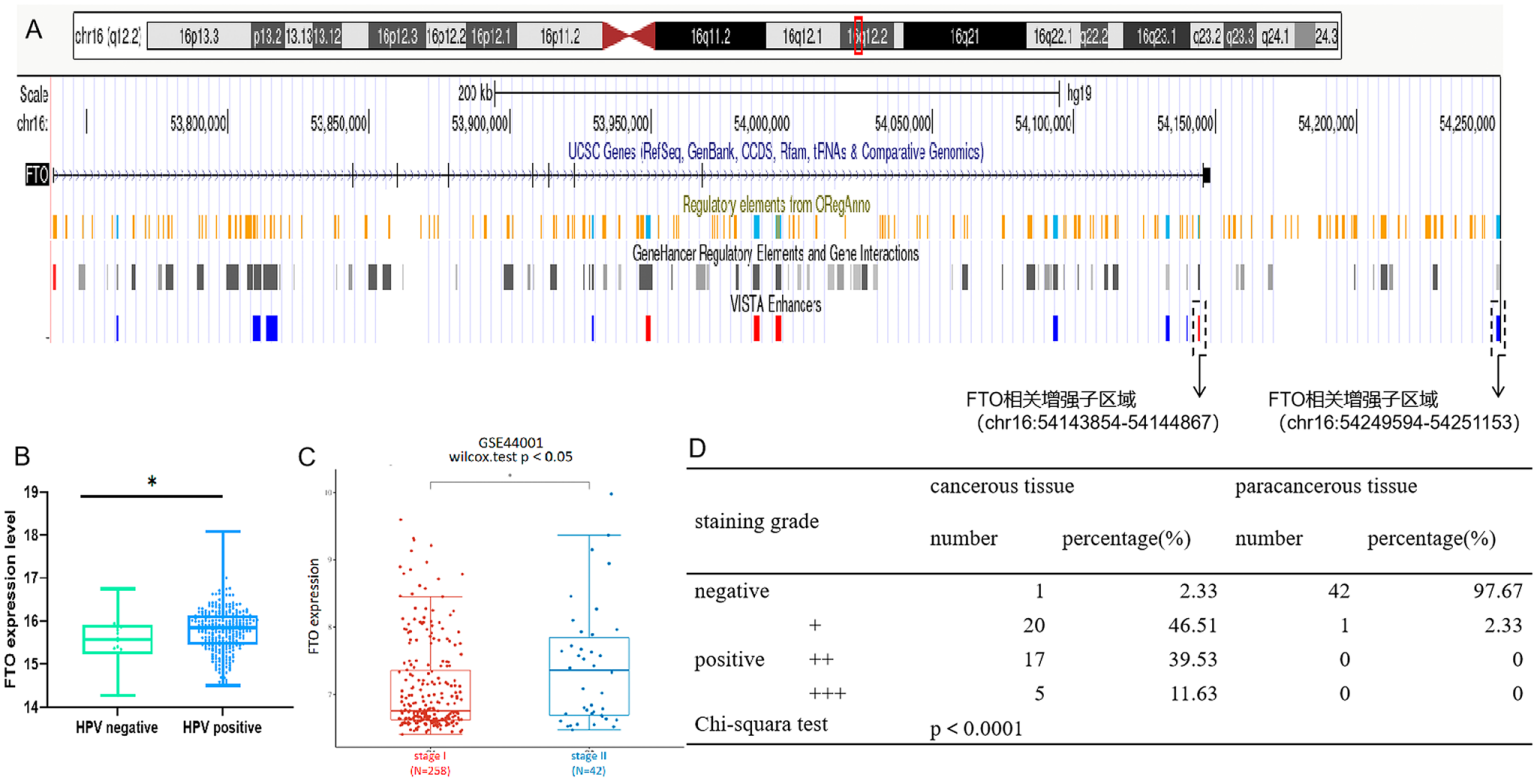
The high level of FTO may be related to HPV intergration in cervical cancer tissue and predicts the late FIGO stage. (A) The UCSC Genome Browser suggests the possible presence of FTO related enhancers may be present in the chr16:54143854–54144867 and chr16:54249594–54251153 (the blue bands in ORegAnno indicate regulatory elements, the black bands in GeneHancer Regulatory Elements indicate enhancers, the red bands in VISTA Enhancer Browser indicate reproducible enhancers, and the blue bands in VISTA Enhancer Browser indicate unreproducible enhancers). (B) The expression levels of FTO in HPV negative cervical cancer patients (*n* = 22) and HPV positive cervical cancer patients (*n* = 281). (C) GSE44001 cohort suggested that the level of FTO expression was higher in FIGO stage II cervical cancer patient than FIGO stage I. (D) IHC was performed on cancer tissues and corresponding paracancerous tissues with FTO as the target protein. The number of positive cells was evaluated by image J. When the proportion of positive cells was less than 25%, the IHC grade was judged as negative, 25% ~ 50% as +, 50% ~ 75% as ++, and more than 75% as +++. Statistical analysis was performed through GraphPad Prism 8.0. The difference between each group of data were calculated through t test, Chi‐square test, or Fisher exact test. The p‐value of < 0.05 was considered statistically significant (*p  <  0.05).

According to the published whole genome sequencing and high‐throughput viral integration detection (HIVID) results, human papillomavirus (HPV) integration sites were identified in clinical samples and HPV integration (integrated position: chr16‐54275001, chr16‐54291966) can affect the expression of FTO gene [9]. The expression of genes is intricately governed by regulatory elements, including enhancers, promoters, and so forth [41]. Enhancers, which are modified by histones histone H3 lysine 27 acetylation (H3K27ac) and histone H3 lysine 4 monomethylation (H3K4me1), can recruit transcription factors, RNA polymerase, and regulatory protein complexes and promote the transcription of target genes [42, 43]. According to the UCSC Genome Browser, there may existed FTO promoters in the region chr16:54143854–54144867 (named as hs52 by VISTA Enhancers) and chr16:54249594–54251153 (named as hs166 by VISTA Enhancers) (Figure 1A) [44]. Based on TCGA‐CESC cohort, the HPV positive patients had higher FTO levels than HPV negative patients (Figure 1B and Table S2). Therefore, we suspect that HPV integration may enhance FTO expression through enhancers, but this needs to be verified by more experiments. In order to systematically study the role of FTO in cervical cancer, we searched GSE44001 data and found that FTO was significantly up‐regulated in FIGO stage II cervical cancer patients, compared with stage I (*p* < 0.05, Figure 1C) [45]. IHC assay was used to detect the expression of FTO protein in forty‐three pairs of FIGO stage II cervical cancer tissues and paracancerous tissues. In the 43 cancerous tissues, one (2.33%) was classified as negative, 20 (46.51%) as +, 17 (39.53%) as ++, and 5 (11.63%) as +++. Besides, there were 42 (97.67%) regarded as negative and one (2.33%) regarded as + in these 43 paracancerous tissues (Figure 1D and Figure S1). The results suggested that the FTO level of cancerous tissue was significantly higher than that of paracancerous tissue (*p* < 0.0001). Given all of that, the increase in FTO may be related to HPV integration and predicts the late FIGO stage in cervical cancer.

### 3.2 FTO Facilitates Cervical Cancer Cell Proliferation and Metastasis In Vitro

**FIGURE 2:**
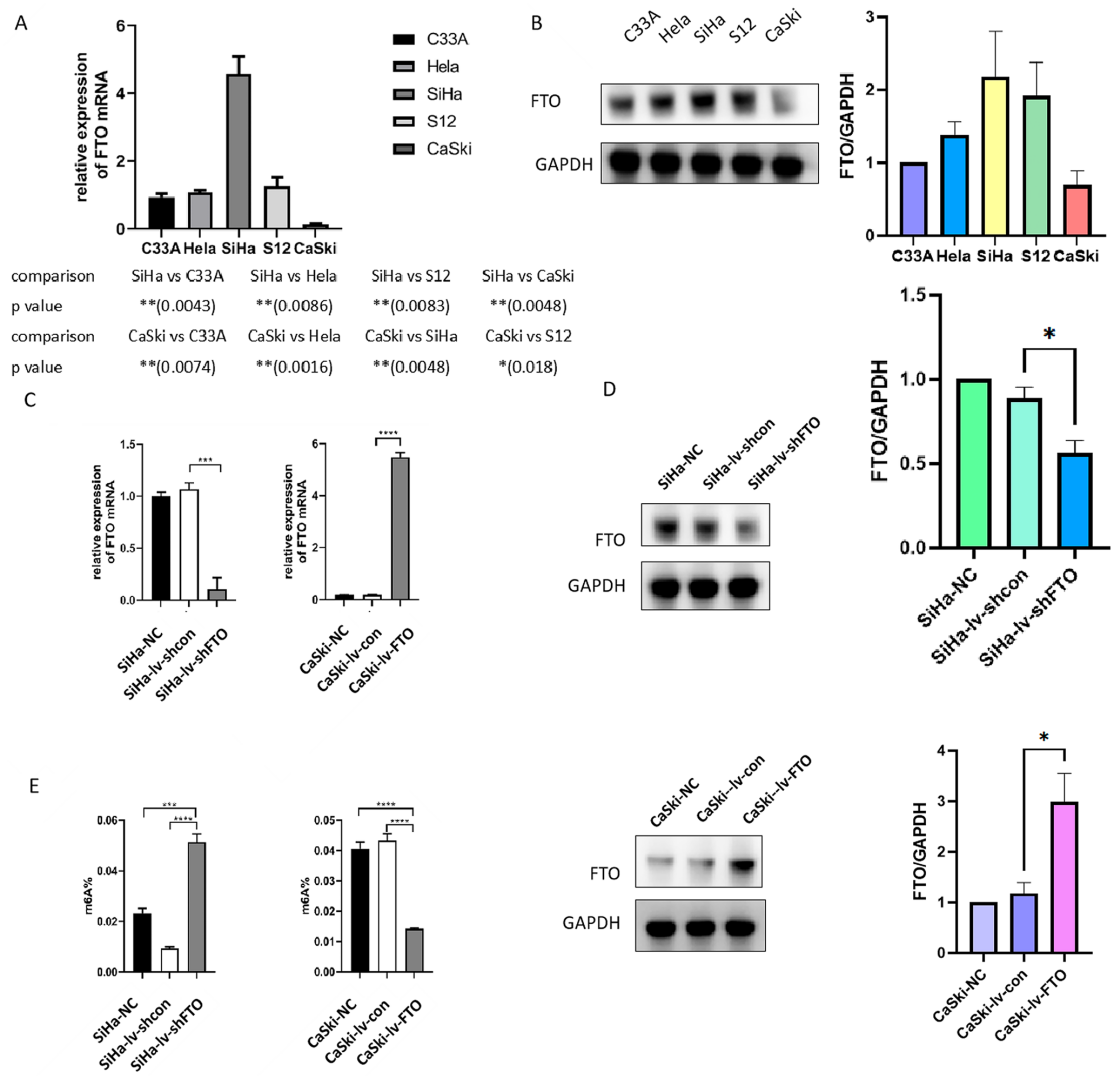
The expression level of FTO can effect the m6A modification in cervical cancer cells. (A) The rt‐qPCR results showed that the post‐transcriptional level of FTO in Siha cells was significantly higher than that in other four cells, while Caski cells was the lowest. (B) The western blot results showed that FTO protein in Siha cells was the highest, while FTO protein in Caski cells was the lowest. (C–E) After virus transfection, the FTO mRNA levels, FTO protein levels, and m6A modification levels were detected. Statistical analysis was performed through GraphPad Prism 8.0. The difference between each group of data were calculated through t test, Chi‐square test, or Fisher exact test. The p‐value of < 0.05 was considered statistically significant (*p  <  0.05; **p  <  0.01; ***p  <  0.001; ****p  <  0.0001).

In order to further confirm the effects of FTO on m6A modification, we respectively constructed cervical cancer cells with FTO knockdown or overexpression. Among C33A, Hela, SiHa, S12, and CaSki cells, the expression level of FTO in SiHa cells was significantly higher than that in other cervical cancer cells, while the expression level of CaSki cells was the lowest (Figure 2A,B). Therefore, we established the FTO knockdown lentivirus infected SiHa cells (SiHa‐lv‐shFTO) and control virus infected SiHa cells (SiHa‐lv‐shcon). Correspondingly, FTO‐overexperssed CaSki cells and control CaSki cells (CaSki‐lv‐FTO and CaSki‐lv‐con) were established. We further detected the FTO expression levels and m6A modification levels in above cells (Figure 2C–E). And the results exemplified that the upregulation of FTO can reduce m6A modification level in cervical cancer cells.

**FIGURE 3:**
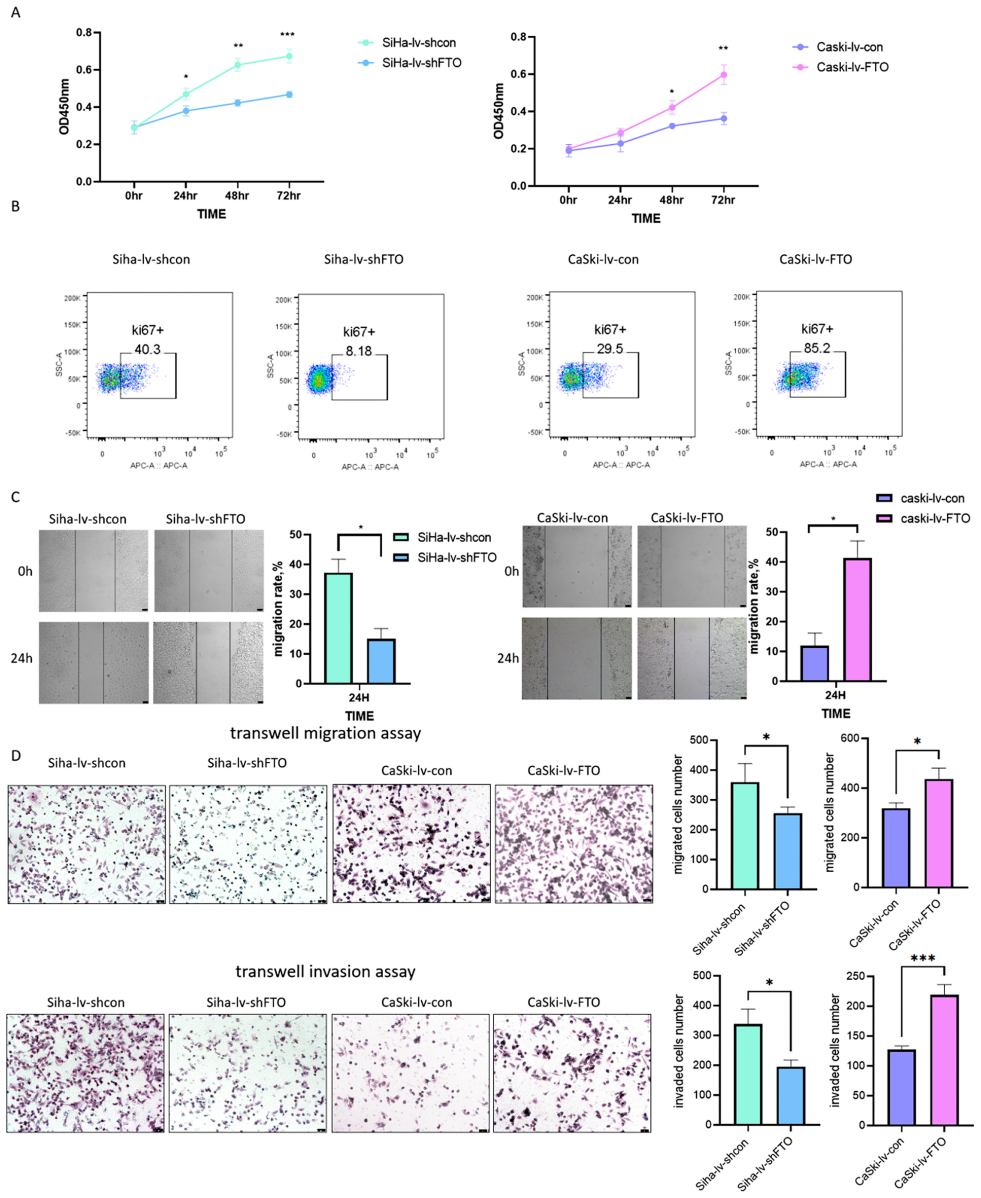
The effect of FTO on the proliferation and metastasis of cervical cancer cells was verified in vitro. (A) CCK8 assay revealed the proliferation activity at 0, 24, 48, and 72 h in cervical cancer cells with different FTO levels, and knockdown of FTO inhibited cell proliferation. (B) Ki67 assay revealed that the proliferation rate of cervical cancer cells with different FTO levels. (C) Wound healing assay revealed the percentage of healing distance at 24 h, and knockdown of FTO inhibited cell migration (the bar represented 50 μm). (D) Transwell migration assay and invasion assay displayed knockdown of FTO inhibited cell migration and invasion (the bar represented 50 μm). Statistical analysis was performed through GraphPad Prism 8.0. The difference between each group of data were calculated through t test, Chi‐square test, or Fisher exact test. The p‐value of < 0.05 was considered statistically significant (*p  <  0.05; **p <  0.01; ***p  <  0.001).

To explore the role of FTO in cervical cancer, CCK8 assay, Ki67 assay, wound healing assay, and transwell assay were performed. The CCK8 assay and Ki67 assay showed that cervical cancer cells grew relatively slowly after FTO down‐regulation, in contrast, FTO overexpression facilitated the proliferative ability (Figure 3A,B). The wound healing assay revealed that knockdown of FTO decreased metastatic ability, while overexpression of FTO accelerated metastatic ability (Figure 3C). Consistent with the results above, in the transwell migration assay and transwell invasion assay, the penetrated cells numbers of SiHa‐lv‐shFTO cells were less than those of SiHa‐lv‐shcon cells, and the penetrated CaSki‐lv‐FTO cells were more than the penetrated CaSki‐lv‐con cells (Figure 3D). These data indicated that FTO promoted cell proliferation, migration, and invasion ability in the cervical cancer cells in vitro.

### 3.3 FTO Promotes the Growth and Metastasis of Cervical Carcinoma Cells In Vivo

**FIGURE 4:**
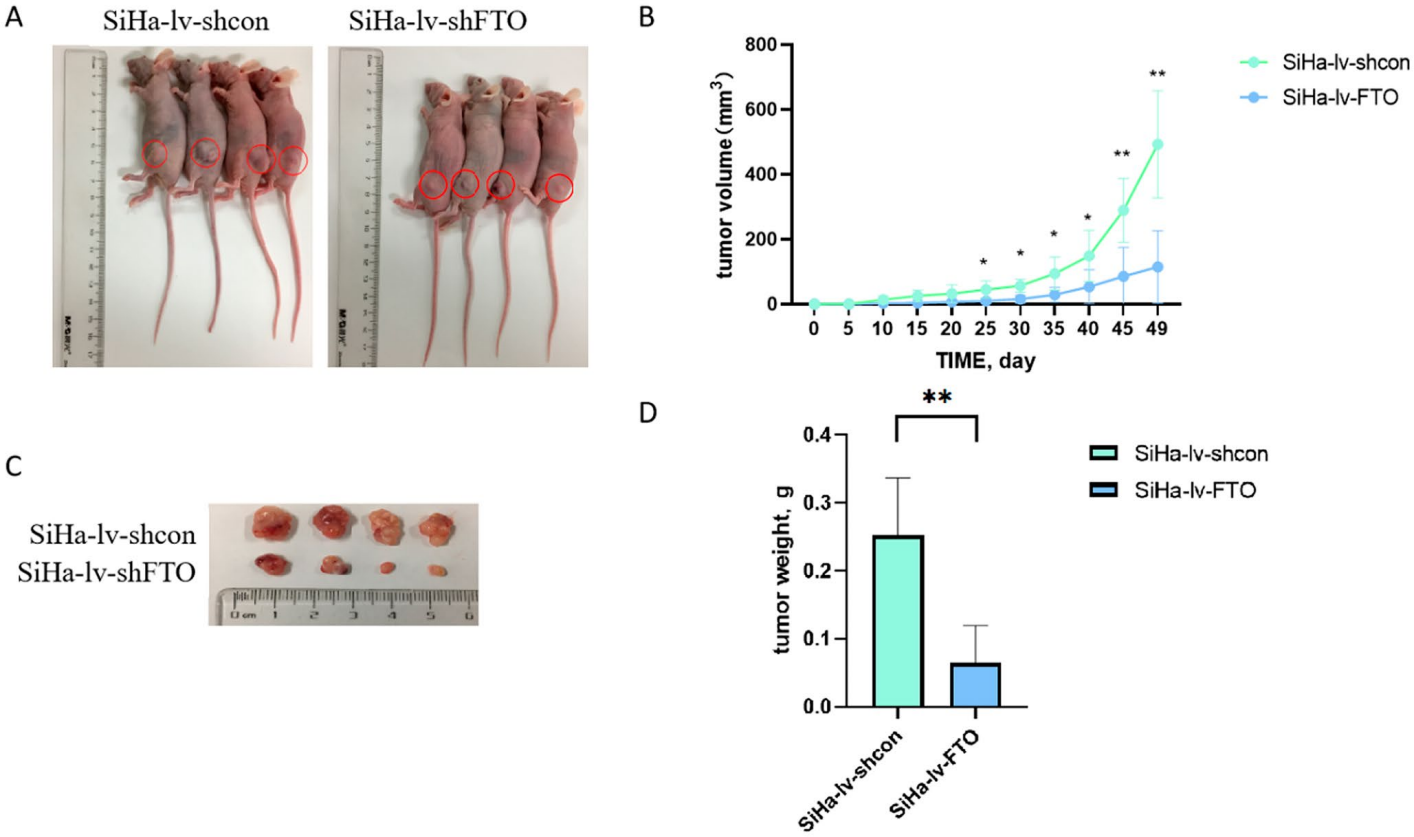
The effect of FTO on the proliferation ability of cervical cancer cells was verified by the subcutaneous tumor model. (A) The pictures of the subcutaneous tumor model in SiHa‐lv‐shcon group and SiHa‐lv‐shFTO group. (B) The curve of subcutaneous tumor volume and time showed that subcutaneous tumors established by SiHa‐lv‐shFTO cells grew significantly slower. (C) The volume of subcutaneous tumor in SiHa‐lv‐shFTO group was less than SiHa‐lv‐shcon group. (D) The weight of subcutaneous tumor in SiHa‐lv‐shFTO group was lighter than SiHa‐lv‐shcon group. Statistical analysis was performed through GraphPad Prism 8.0. The difference between each group of data were calculated through t test, Chi‐square test, or Fisher exact test. The p‐value of < 0.05 was considered statistically significant (*p  < 0.05; **p  <  0.01).

To verify the effect of FTO in vivo, we injected SiHa‐lv‐shcon or SiHa‐lv‐shFTO cells subcutaneously in nude mice to construct subcutaneous tumor model (Figure 4A). Subcutaneous tumors established by SiHa‐lv‐shFTO cells grew significantly slower than that of SiHa‐lv‐shcon cells (Figure 4B). And compared with SiHa‐lv‐shcon group, the tumor volume and tumor weight were dramatically less in SiHa‐lv‐shFTO group (Figure 4C,D). Taken together, these results suggested that FTO downregulation had an inhibitory effect on tumor growth in vivo.

**FIGURE 5:**
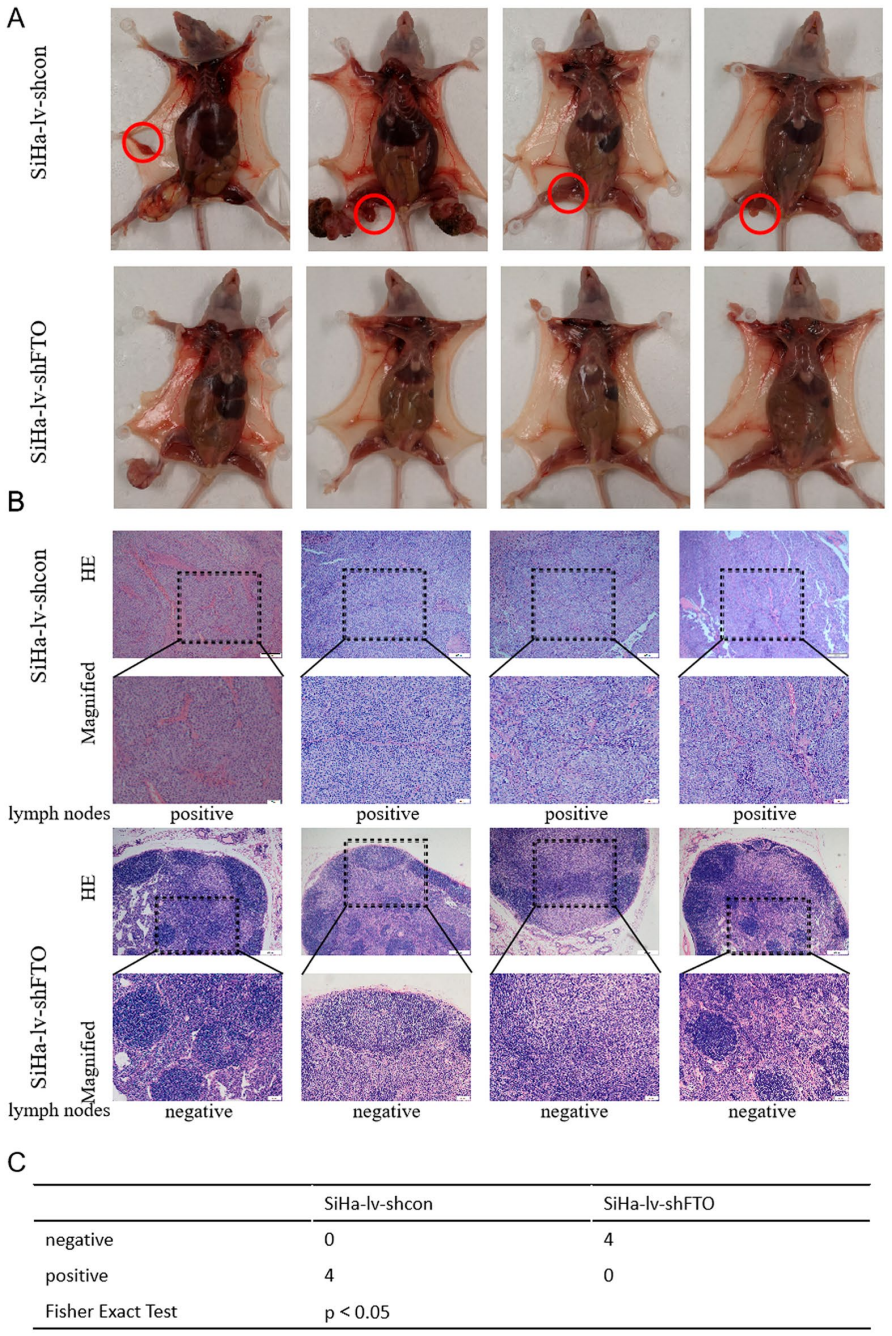
The effect of FTO on the metastatic ability of cervical cancer cells was verified by lymph node metastasis model. (A) The pictures of the lymph node metastasis model in SiHa‐lv‐shcon group and SiHa‐lv‐shFTO group. (B) Hematoxylin–eosin staining results of lymph nodes of nude mice in SiHa‐lv‐shcon group and SiHa‐lv‐shFTO group (the bar represented 200 μm). (C) Statistical analysis showed that nude mice in SiHa‐lv‐shFTO group had less lymph node metastasis than SiHa‐lv‐shcon group.

To construct lymph node metastasis models, SiHa‐lv‐shcon or SiHa‐lv‐shFTO cells were slowly injected into the foot pads in nude mice (Figure 5A). In SiHa‐lv‐shcon group, four nude mice had lymph node metastasis. And in SiHa‐lv‐shFTO group, no lymph node metastasis was observed (Figure 5B,C). The above findings suggested that FTO promoted tumor metastasis in cervical cancer in vivo.

### 3.4 Merip‐Seq Determines the Target Genes of FTO

**FIGURE 6:**
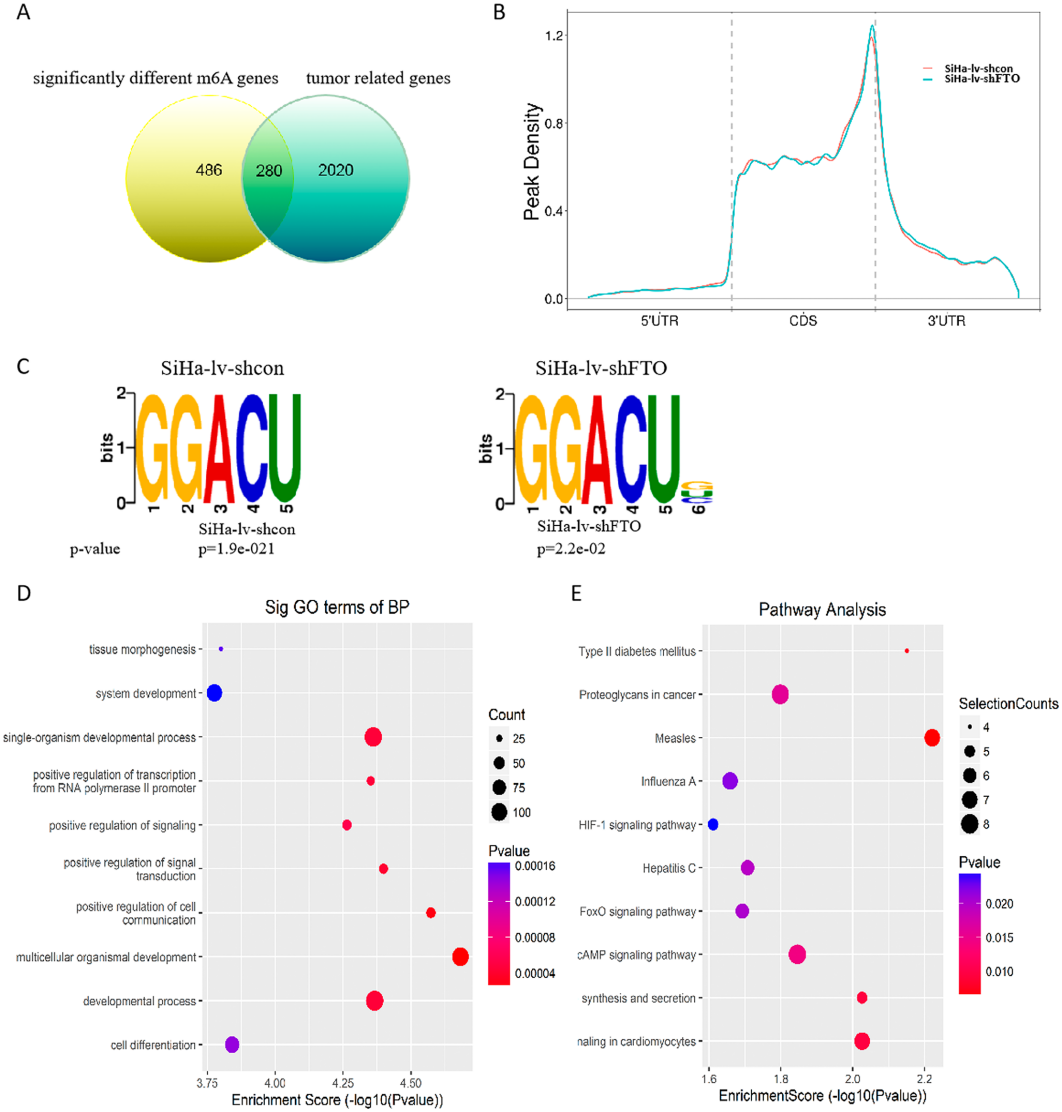
The Merip‐seq of SiHa‐lv‐shcon and SiHa‐lv‐shFTO cells. (A) Venn diagram of significantly different m6A modification genes and tumor related genes. (B) MetaPlotR was used to draw the distribution map of methylation peaks on metagene of SiHa‐lv‐shcon and SiHa‐lv‐shFTO cells. (C) Motif analysis showed that the sequences of the first 2000 peaks (50 bp on each side of the vertex) with the largest enrichment factor of each group were scanned by dreme to find the most significant motif sequence. (D, E) The significant GO term of BP and KEGG pathway analysis between SiHa‐lv‐shcon and SiHa‐lv‐shFTO cells.

To investigate the regulatory mechanism of FTO in cervical cancer malignancy, the RNA from SiHa‐lv‐shcon and SiHa‐lv‐shFTO cells was collected to perform Merip‐seq for transcriptome‐wide m6A map. There were 486 genes whose m6A modification levels were significantly higher in SiHa‐lv‐shFTO cells than those in SiHa‐lv‐shcon cells. After intersection with 2020 tumor‐related genes from OnGene database and TSGene database, 280 genes of interest were obtained (Figure 6A) [46, 47]. The density of m6A peaks increased along the transcript in the CDS region (Figure 6B). And the consensus “GGACU” motif was highly enriched within m6A sites in both SiHa‐lv‐shcon cells and SiHa‐lv‐shFTO cells (Figure 6C). Based on the genes with differential m6A modification, GO enrichment analysis assigned these genes of interest to mutiple biological processes, and KEGG enrichment results indicated the top 10 pathways associated with FTO‐dependent m6A RNA methylation, including FoxO signaling pathway (Figure 6D,E and Tables S3 and S4). According to previous research, PIK3R3 works as a crucial regulator of FoxO pathway and promotes the progress of malignant tumors [32, 33, 34, 35, 36, 37, 38]. Therefore, we take PIK3R3 as the breakthrough point to further explore the mechanism of FTO promoting the proliferation and metastasis of cervical cancer.

### 3.5 FTO Promotes Cervical Cancer Malignancy by Up Regulating PIK3R3

**FIGURE 7:**
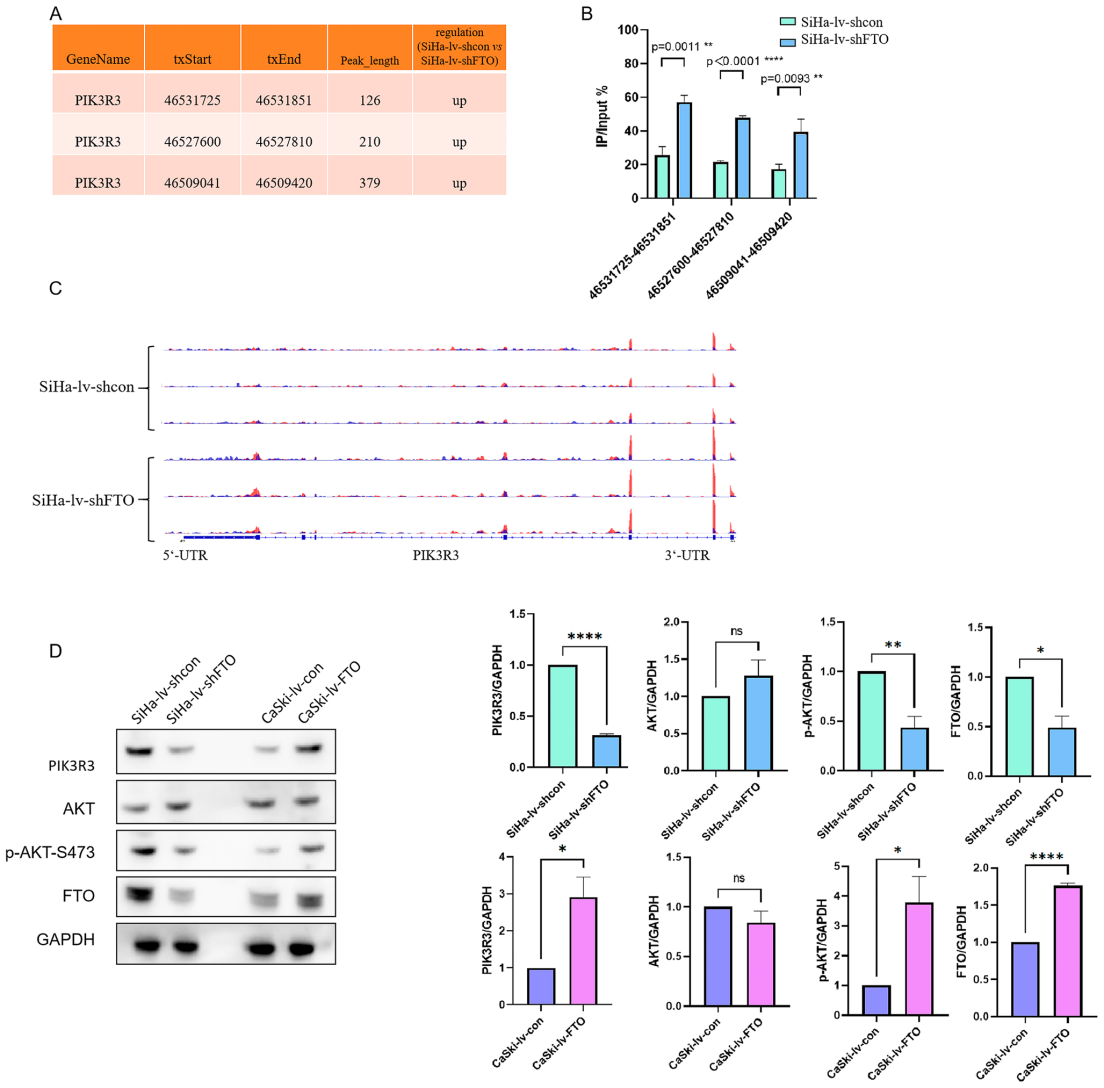
The validation of the effect of FTO on the FoxO pathway. (A) The three m6A modification peaks of PIK3R3 mRNA were detected in SiHa‐lv‐shcon and SiHa‐lv‐shFTO cells. (B, C)Merip‐qPCR and the IGV peak map all displayed the higher m6A modification levels in SiHa‐lv‐shFTO cells. (D) The protein levels of FTO, Akt, p‐Akt, PIK3R3 and GAPDH were detected by western blot in SiHa‐lv‐shcon, SiHa‐lv‐shFTO cells and CaSki‐lv‐con, CaSki‐lv FTO cells. Statistical analysis was performed through GraphPad Prism 8.0. The difference between each group of data were calculated through t test, Chi‐square test, or Fisher exact test. The p‐value of < 0.05 was considered statistically significant (*p  <  0.05; **p  <  0.01; ****p  <  0.0001).

Based on the Merip‐seq, there were three m6A modification sites detected on PIK3R3 mRNA (Figure 7A). And the Merip‐qPCR and the IGV peak map displayed that m6A modification levels of these three sites were all higher in SiHa‐lv‐shFTO cells (Figure 7B,C). Western blot showed that when the expression level of FTO in cervical cancer cells was downregulated, the expressions of PIK3R3 were also decreased, as well as p‐AKT. Correspondingly, when FTO was upregulated, the expression levels of PIK3R3 and p‐AKT were also increased (Figure 7D).

**FIGURE 8:**
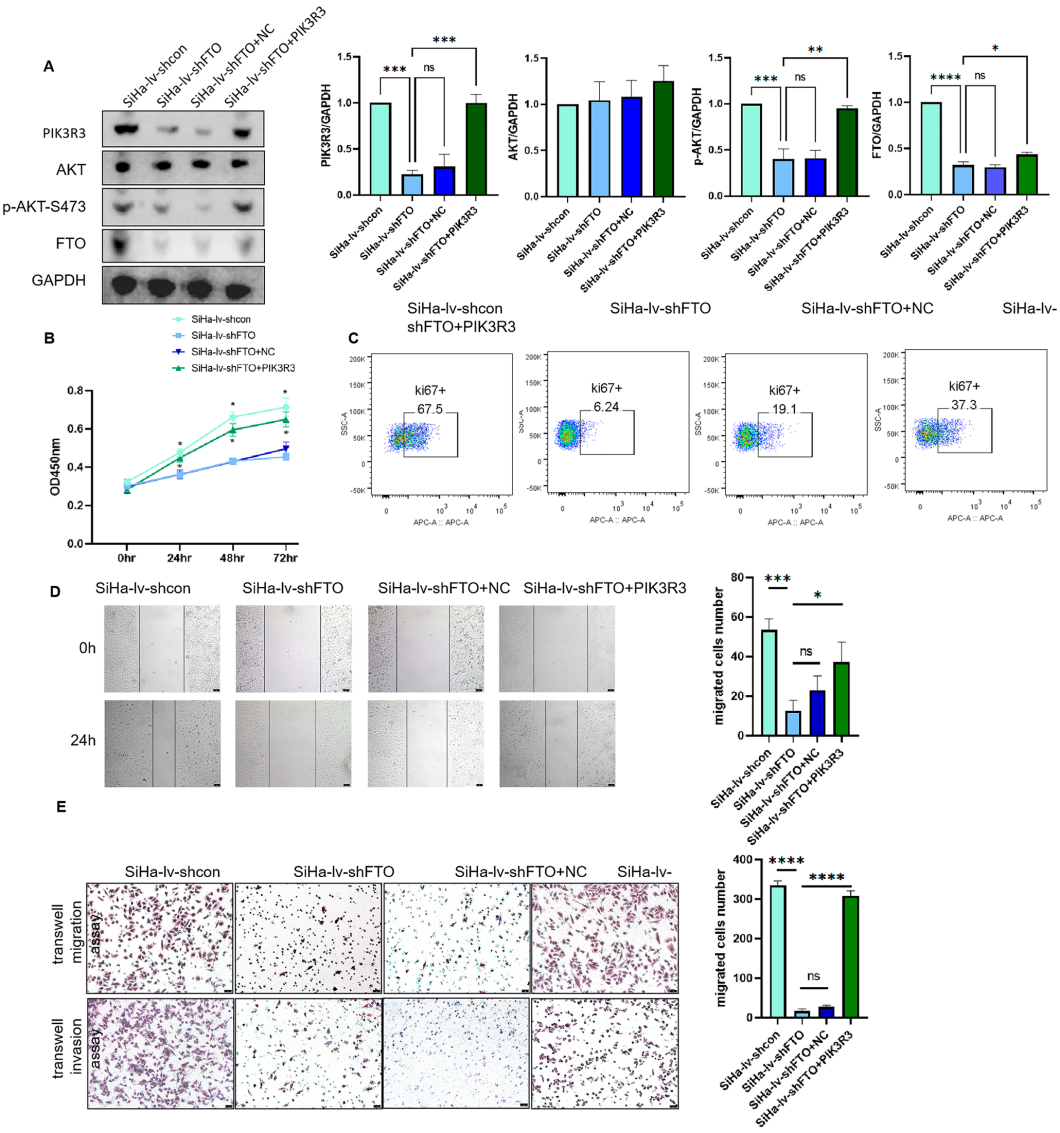
FTO could affect the cervical cancer malignancy through PIK3R3. (A) After PIK3R3 overexpression, the western blot displayed that p‐Akt also increased. (B, C) After PIK3R3 overexpression, CCK8 assay and Ki67 assay showed that proliferation ability increased. (D, E) After PIK3R3 overexpression, wound healing assay, transwell migration, and invasion assay displayed that the migration and invasion ability increased (the bar represented 50 μm). Statistical analysis was performed through GraphPad Prism 8.0. The difference between each group of data were calculated through t test, Chi‐square test, or Fisher exact test. The p‐value of < 0.05 was considered statistically significant (*p  <  0.05; **p  <  0.01; ***p  <  0.001; ****p  <  0.0001).

To further illustrate that PIK3R3 and p‐AKT is regulated by FTO in cervical cancer cells, we transfected control plasmid and PIK3R3 plasmid into SiHa‐lv‐shFTO cells, respectively. Also, the western blot indicated that the upregulation of PIK3R3 can partially neutralize the effect of FTO‐knockdown on p‐AKT in SiHa cells (Figure 8A). Next, we conducted CCK8 assay, Ki67 assay, wound healing assay and transwell assay. Our data verified that PIK3R3 overexpression can partially reverse the inhibitory effect of FTO knockdown on proliferation, migration and invasion of cervical cancer cells (Figure 8B–E). Based on the above results, FTO promoted cervical cancer malignancy by regulating m6A modification of PIK3R3 mRNA.

## 4 Discussion

Recently, more and more evidence shows that malignant tumor progression largely depend on epigenetic regulation. M6A is one of the most common methylation modification, and aberrant m6A modification work as an important tumor promoter. In cervical cancer, the chronic infection of high‐risk HPV is the main cause, thereby affecting the oncogenes or tumor suppressor genes [9]. Hu et al. [9] reported that HPV integration was detected at 16q12 in cervical cancer patients based on HIVID, which affected the gene FTO. Also, the UCSC Genome Browser displayed the enhancers of FTO adjacent to HPV integration region. Based on TCFA‐CESC cohort, the FTO expression level in HPV positive cervical cancer patients is higher than that of HPV negative cervical cancer patients. What's more, FTO is highly expressed in cervical cancer and predicts the late FIGO stage. Thus, it can be seen that, the increase in FTO may be caused by HPV integration in cervical cancer, and it plays a cancer‐promoting role in cervical cancer.

PIK3R3 can encode p55γ to form the p85 in PI3K and trigger the phosphorylation cascade in FoxO pathway. The role of PIK3R3 in several malignant tumors has been confirmed [32, 33, 34, 35, 36, 37, 38]. Yoon C et al. found that the upregulation of PIK3R3 can activate Akt and ERK signals to promote the invasion, migration, and chemoresistance in sarcoma cancer stem‐like cells [32]. Also, Wang G et al. displayed that PIK3R3 promoted colorectal cancer metastasis via SNAI2‐induced epithelial‐to‐mesenchymal transition, suggesting a potential target for metastatic colorectal cancer [38]. Our experiment displayed that FTO can promote proliferation and migration in cervical cancer cells, and upregulation of PIK3R3 can partially neutralize the effect of FTO‐knockdown. All these results suggested that FTO facilitated cervical cancer malignancy through inducing m6A‐demethylation of PIK3R3 mRNA.

Due to the close relationship between m6A modification and malignant tumors, the adjustment of m6A modification may become a new cancer treatment strategy [48, 49, 50, 51, 52]. Su et al. [49] found that R‐2HG, as an FTO inhibitor, showed antitumor activity by targeting FTO/m6A/myc/CEBPA signals in leukocytes. Phan et al. [52] displayed that FTO inhibitor CS1 and lentivirus‐mediated FTO knockout can suppress the proliferation of colorectal cancer cells in vivo and in vitro, and CS1 induced arrest of the cell cycle in the G2/M phase through downregulation of CDC25C, further triggering apoptosis. Our experimental results showed that FTO is highly expressed in cervical cancer and predicts the late FIGO stage. More importantly, we found that the FTO knockdown can inhibit the proliferation and metastasis of cervical cancer cells in vitro and vivo. Therefore, the adjustment of m6A modification may serve as an essential promising therapeutic target for cervical cancer.

**FIGURE 9:**
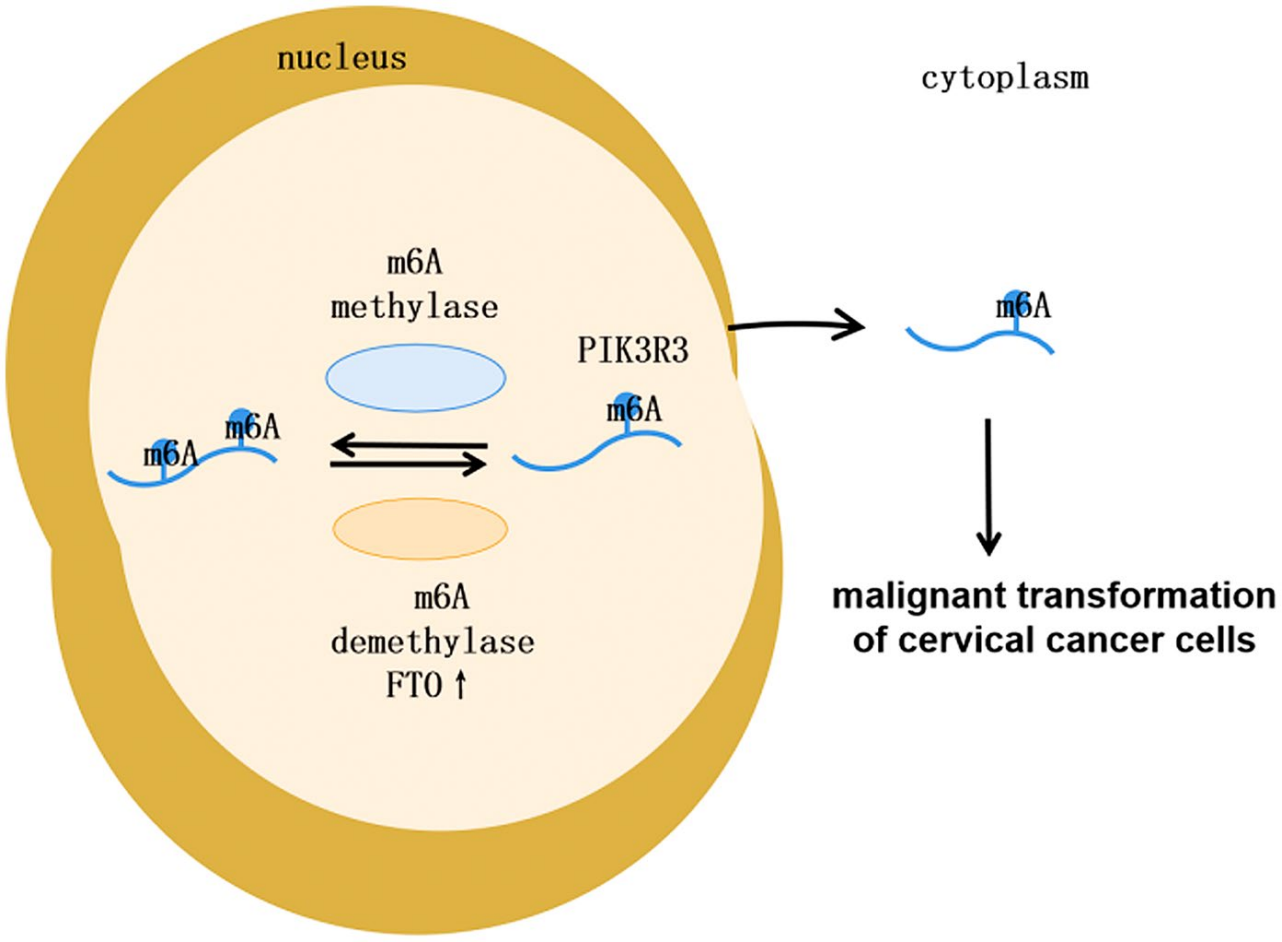
The schematic diagram of biological function and potential mechanisms for FTO in cervical cancer.

Taking our findings together, the upregulation of m6A demethylase FTO decreases m6A modification of PIK3R3 mRNA, which increases PIK3R3 levels and activates FoxO pathway, thus facilitating cervical cancer malignancy (Figure 9).

## Author Contributions

**Bingxin Chen:** conceptualization (lead), data curation (equal), formal analysis (equal), funding acquisition (supporting), investigation (lead), methodology (lead), project administration (supporting), resources (equal), software (lead), validation (equal), visualization (equal), writing – original draft (lead). **Liming Wang:** data curation (equal), formal analysis (equal), investigation (equal), resources (equal), software (equal), validation (equal), visualization (equal). **Xiaomin Li:** data curation (equal), formal analysis (equal), software (equal). **Ci Ren:** formal analysis (equal), investigation (equal), visualization (equal). **Chun Gao:** investigation (equal), software (equal). **Wencheng Ding:** data curation (equal), resources (equal). **Hui Wang:** conceptualization (equal), funding acquisition (lead), methodology (equal), project administration (lead), supervision (lead), writing – review and editing (lead).

## Ethics Statement

The research project was approved by the Ethics Committee of Tongji Hospital, Huazhong University of Science and Technology (TJ‐IRB20210207 and TJH‐202010008).

## Conflicts of Interest

The authors declare no conflicts of interest.

## Supporting information


Data S1.

## Data Availability

The data are available from the corresponding author on reasonable request.
