# Supplementary material for: FTO Facilitates Cervical Cancer Malignancy Through Inducing m6A‐Demethylation of PIK3R3 mRNA
Source: Cancer Med. 2024 Dec 18;13(24):e70507. doi: 10.1002/cam4.70507 (PMC11653219; doi:10.1002/cam4.70507)
Supplement: Supplementary file 1 — Data S1. [file CAM4-13-e70507-s001.docx]

**Supporting Information**

FTO facilitates cervical cancer malignancy through inducing m6A-demethylation of PIK3R3 mRNA

Bingxin Chen^1, 2^, Liming Wang^1, 2^, Xiaomin Li^1^, Ci Ren^1^, Chun Gao^1^, Wencheng Ding^1^, Hui Wang^1, 2^*

1 Department of Obstetrics and Gynecology, Tongji Hospital, Tongji Medical College, Huazhong University of Science and Technology, Wuhan, Hubei, 430000, China.

2 Department of Gynecologic Oncology, Women's Hospital, Zhejiang University School of Medicine, Hangzhou, 310006, China.

*Corresponding author: Hui Wang, Professor

Women's Hospital, Zhejiang University School of Medicine, Hangzhou, 310006, China

Email: [huit71@sohu.com](mailto:huit71@sohu.com)

**Supplementary Figure 1:**

IHC results in cancer tissues and corresponding paracancerous tissues. The number of positive cells was evaluated by image J. When the proportion of positive cells was less than 25%, the IHC grade was judged as negative, 25 ~ 50% as +, 50 ~ 75% as + +, and more than 75% as + + +.


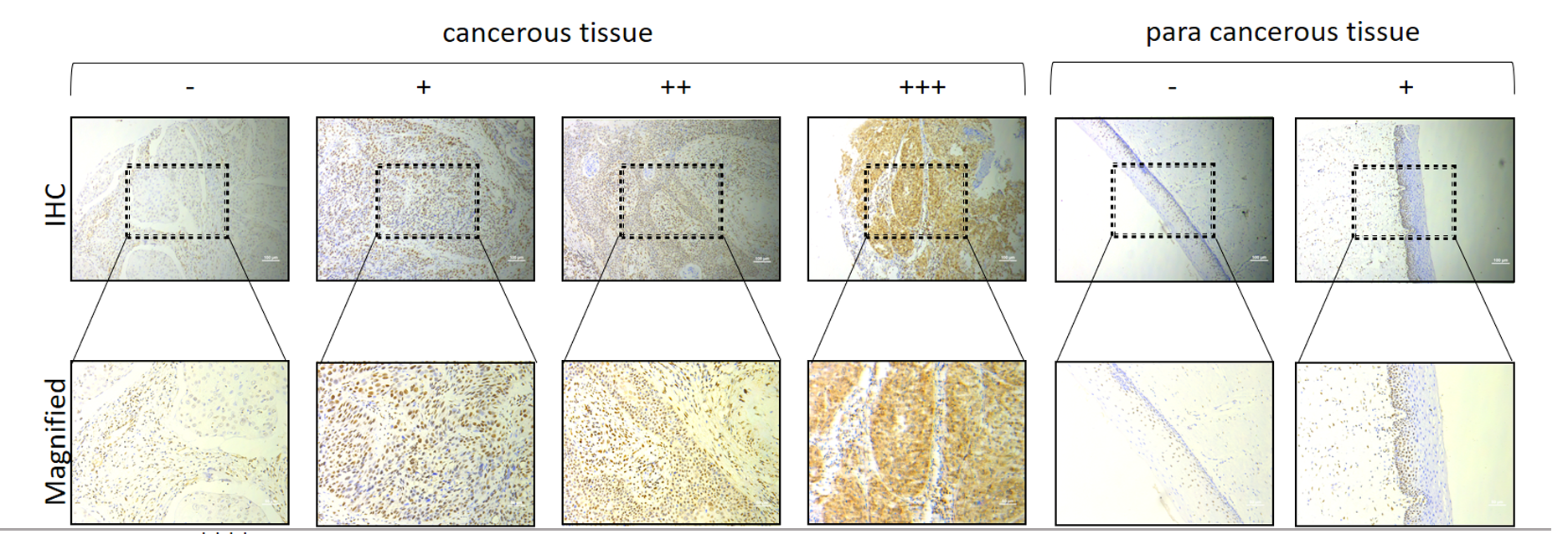


**Supplementary Table 1:**

The clinical pathological characters of included cervical cancer in the tissue microarray.

| Case.No | diagnosis | sex | grade | Case.No | diagnosis | sex | grade |
| --- | --- | --- | --- | --- | --- | --- | --- |
| 1 | cervical cancer tissue | female | grade II | 1 | paracancerous tissue | female | - |
| 2 | cervical cancer tissue | female | grade II | 2 | paracancerous tissue | female | - |
| 3 | cervical cancer tissue | female | grade II | 3 | paracancerous tissue | female | - |
| 4 | cervical cancer tissue | female | grade II | 4 | paracancerous tissue | female | - |
| 5 | cervical cancer tissue | female | grade II | 5 | paracancerous tissue | female | - |
| 6 | cervical cancer tissue | female | grade II | 6 | paracancerous tissue | female | - |
| 7 | cervical cancer tissue | female | grade II | 7 | paracancerous tissue | female | - |
| 8 | cervical cancer tissue | female | grade II | 8 | paracancerous tissue | female | - |
| 9 | cervical cancer tissue | female | grade II | 9 | paracancerous tissue | female | - |
| 10 | cervical cancer tissue | female | grade II | 10 | paracancerous tissue | female | - |
| 11 | cervical cancer tissue | female | grade II | 11 | paracancerous tissue | female | - |
| 12 | cervical cancer tissue | female | grade II | 12 | paracancerous tissue | female | - |
| 13 | cervical cancer tissue | female | grade II | 13 | paracancerous tissue | female | - |
| 14 | cervical cancer tissue | female | grade II | 14 | paracancerous tissue | female | - |
| 15 | cervical cancer tissue | female | grade II | 15 | paracancerous tissue | female | - |
| 16 | cervical cancer tissue | female | grade II | 16 | paracancerous tissue | female | - |
| 17 | cervical cancer tissue | female | grade II | 17 | paracancerous tissue | female | - |
| 18 | cervical cancer tissue | female | grade II | 18 | paracancerous tissue | female | - |
| 19 | cervical cancer tissue | female | grade II | 19 | paracancerous tissue | female | - |
| 20 | cervical cancer tissue | female | grade II | 20 | paracancerous tissue | female | - |
| 21 | cervical cancer tissue | female | grade II | 21 | paracancerous tissue | female | - |
| 22 | cervical cancer tissue | female | grade II | 22 | paracancerous tissue | female | - |
| 23 | cervical cancer tissue | female | grade II | 23 | paracancerous tissue | female | - |
| 24 | cervical cancer tissue | female | grade II | 24 | paracancerous tissue | female | - |
| 25 | cervical cancer tissue | female | grade II | 25 | paracancerous tissue | female | - |
| 26 | cervical cancer tissue | female | grade II | 26 | paracancerous tissue | female | - |
| 27 | cervical cancer tissue | female | grade II | 27 | paracancerous tissue | female | - |
| 28 | cervical cancer tissue | female | grade II | 28 | paracancerous tissue | female | - |
| 29 | cervical cancer tissue | female | grade II | 29 | paracancerous tissue | female | - |
| 30 | cervical cancer tissue | female | grade II | 30 | paracancerous tissue | female | - |
| 31 | cervical cancer tissue | female | grade II | 31 | paracancerous tissue | female | - |
| 32 | cervical cancer tissue | female | grade II | 32 | paracancerous tissue | female | - |
| 33 | cervical cancer tissue | female | grade II | 33 | paracancerous tissue | female | - |
| 34 | cervical cancer tissue | female | grade II | 34 | paracancerous tissue | female | - |
| 35 | cervical cancer tissue | female | grade II | 35 | paracancerous tissue | female | - |
| 36 | cervical cancer tissue | female | grade II | 36 | paracancerous tissue | female | - |
| 37 | cervical cancer tissue | female | grade II | 37 | paracancerous tissue | female | - |
| 38 | cervical cancer tissue | female | grade II | 38 | paracancerous tissue | female | - |
| 39 | cervical cancer tissue | female | grade II | 39 | paracancerous tissue | female | - |
| 40 | cervical cancer tissue | female | grade II | 40 | paracancerous tissue | female | - |
| 41 | cervical cancer tissue | female | grade II | 41 | paracancerous tissue | female | - |
| 42 | cervical cancer tissue | female | grade II | 42 | paracancerous tissue | female | - |
| 43 | cervical cancer tissue | female | grade II | 43 | paracancerous tissue | female | - |

**Supplementary Table 2:**

The TCGA ID of HPV positive cervical cancer patients and HPV negative cervical cancer patients.

| HPV status | Number of patients | TCGA ID |
| --- | --- | --- |
| positive | 281 | TCGA-4J-AA1J-01A, TCGA-BI-A0VR-01A, TCGA-BI-A0VS-01A, TCGA-BI-A20A-01A, TCGA-C5-A0TN-01A, TCGA-C5-A1BE-01B, TCGA-C5-A1BF-01B, TCGA-C5-A1BI-01B, TCGA-C5-A1BJ-01A, TCGA-C5-A1BK-01B, TCGA-C5-A1BL-01A, TCGA-C5-A1BM-01A, TCGA-C5-A1BN-01B, TCGA-C5-A1BQ-01C, TCGA-C5-A1M5-01A, TCGA-C5-A1M6-01A, TCGA-C5-A1M7-01A, TCGA-C5-A1M8-01A, TCGA-C5-A1M9-01A, TCGA-C5-A1ME-01A, TCGA-C5-A1MF-01A, TCGA-C5-A1MH-01A, TCGA-C5-A1MI-01A, TCGA-C5-A1MJ-01A, TCGA-C5-A1MK-01A, TCGA-C5-A1ML-01A, TCGA-C5-A1MN-01A, TCGA-C5-A1MP-01A, TCGA-C5-A1MQ-01A, TCGA-C5-A2LS-01A, TCGA-C5-A2LV-01A, TCGA-C5-A2LX-01A, TCGA-C5-A2LY-01A, TCGA-C5-A2LZ-01A, TCGA-C5-A2M1-01A, TCGA-C5-A2M2-01A, TCGA-C5-A3HD-01B, TCGA-C5-A3HE-01A, TCGA-C5-A3HF-01A, TCGA-C5-A3HL-01A, TCGA-C5-A7CG-01A, TCGA-C5-A7CH-01A, TCGA-C5-A7CJ-01A, TCGA-C5-A7CK-01A, TCGA-C5-A7CL-01A, TCGA-C5-A7CM-01A, TCGA-C5-A7CO-01A, TCGA-C5-A7UC-01A, TCGA-C5-A7UE-01A, TCGA-C5-A7UH-01A, TCGA-C5-A7UI-01A, TCGA-C5-A7X3-01A, TCGA-C5-A7X5-01A, TCGA-C5-A7X8-01A, TCGA-C5-A7XC-01A, TCGA-C5-A8XH-01A, TCGA-C5-A8XI-01A, TCGA-C5-A8XJ-01A, TCGA-C5-A8XK-01A, TCGA-C5-A8YR-01A, TCGA-C5-A8YT-01A, TCGA-C5-A8ZZ-01A, TCGA-C5-A901-01A, TCGA-C5-A902-01A, TCGA-C5-A905-01A, TCGA-C5-A907-01A, TCGA-DG-A2KH-01A, TCGA-DG-A2KJ-01A, TCGA-DG-A2KK-01A, TCGA-DG-A2KL-01A, TCGA-DG-A2KM-01A, TCGA-DR-A0ZL-01A, TCGA-DR-A0ZM-01A, TCGA-DS-A0VK-01A, TCGA-DS-A0VL-01A, TCGA-DS-A0VM-01A, TCGA-DS-A0VN-01A, TCGA-DS-A1O9-01A, TCGA-DS-A1OA-01A, TCGA-DS-A1OB-01A, TCGA-DS-A1OC-01A, TCGA-DS-A1OD-01A, TCGA-DS-A5RQ-01A, TCGA-DS-A7WF-01A, TCGA-DS-A7WH-01A, TCGA-DS-A7WI-01A, TCGA-EA-A1QS-01A, TCGA-EA-A1QT-01A, TCGA-EA-A3HQ-01A, TCGA-EA-A3HR-01A, TCGA-EA-A3HS-01A, TCGA-EA-A3HT-01A, TCGA-EA-A3HU-01A, TCGA-EA-A3QD-01A, TCGA-EA-A3QE-01A, TCGA-EA-A3Y4-01A, TCGA-EA-A411-01A, TCGA-EA-A439-01A, TCGA-EA-A43B-01A, TCGA-EA-A44S-01A, TCGA-EA-A50E-01A, TCGA-EA-A5FO-01A, TCGA-EA-A5O9-01A, TCGA-EA-A5ZD-01A, TCGA-EA-A5ZE-01A, TCGA-EA-A5ZF-01A, TCGA-EA-A6QX-01A, TCGA-EA-A78R-01A, TCGA-EA-A97N-01A, TCGA-EK-A2GZ-01A, TCGA-EK-A2H0-01A, TCGA-EK-A2H1-01A, TCGA-EK-A2IP-01A, TCGA-EK-A2IR-01A, TCGA-EK-A2PG-01A, TCGA-EK-A2PI-01A, TCGA-EK-A2PK-01A, TCGA-EK-A2PL-01A, TCGA-EK-A2R7-01A, TCGA-EK-A2R8-01A, TCGA-EK-A2R9-01A, TCGA-EK-A2RA-01A, TCGA-EK-A2RB-01A, TCGA-EK-A2RC-01A, TCGA-EK-A2RE-01A, TCGA-EK-A2RJ-01A, TCGA-EK-A2RK-01A, TCGA-EK-A2RL-01A, TCGA-EK-A2RM-01A, TCGA-EK-A2RN-01A, TCGA-EK-A2RO-01A, TCGA-EK-A3GJ-01A, TCGA-EK-A3GK-01A, TCGA-EK-A3GM-01A, TCGA-EK-A3GN-01A, TCGA-EX-A1H5-01A, TCGA-EX-A1H6-01B, TCGA-EX-A3L1-01A, TCGA-EX-A449-01A, TCGA-EX-A69L-01A, TCGA-EX-A69M-01A, TCGA-EX-A8YF-01A, TCGA-FU-A23K-01A, TCGA-FU-A23L-01A, TCGA-FU-A2QG-01A, TCGA-FU-A3HY-01A, TCGA-FU-A3NI-01A, TCGA-FU-A3TQ-01A, TCGA-FU-A3TX-01A, TCGA-FU-A3WB-01A, TCGA-FU-A3YQ-01A, TCGA-FU-A40J-01A, TCGA-FU-A5XV-01A, TCGA-FU-A770-01A, TCGA-GH-A9DA-01A, TCGA-HG-A2PA-01A, TCGA-HM-A3JJ-01A, TCGA-HM-A3JK-01A, TCGA-HM-A4S6-01A, TCGA-IR-A3L7-01A, TCGA-IR-A3LB-01A, TCGA-IR-A3LC-01A, TCGA-IR-A3LF-01A, TCGA-IR-A3LH-01A, TCGA-IR-A3LI-01A, TCGA-IR-A3LK-01A, TCGA-IR-A3LL-01A, TCGA-JW-A5VG-01A, TCGA-JW-A5VJ-01A, TCGA-JW-A5VL-01A, TCGA-JW-A69B-01A, TCGA-JW-A852-01A, TCGA-JW-AAVH-01A, TCGA-JX-A3PZ-01A, TCGA-JX-A3Q0-01A, TCGA-JX-A3Q8-01A, TCGA-JX-A5QV-01A, TCGA-LP-A4AU-01A, TCGA-LP-A4AV-01A, TCGA-LP-A4AW-01A, TCGA-LP-A4AX-01A, TCGA-LP-A5U2-01A, TCGA-LP-A5U3-01A, TCGA-LP-A7HU-01A, TCGA-MA-AA3W-01A, TCGA-MA-AA3X-01A, TCGA-MA-AA3Y-01A, TCGA-MA-AA3Z-01A, TCGA-MA-AA41-01A, TCGA-MA-AA42-01A, TCGA-MA-AA43-01A, TCGA-MU-A51Y-01A, TCGA-MU-A5YI-01A, TCGA-MU-A8JM-01A, TCGA-MY-A5BD-01A, TCGA-MY-A5BE-01A, TCGA-MY-A5BF-01A, TCGA-MY-A913-01A, TCGA-PN-A8MA-01A, TCGA-Q1-A5R1-01A, TCGA-Q1-A5R2-01A, TCGA-Q1-A5R3-01A, TCGA-Q1-A6DT-01A, TCGA-Q1-A6DV-01A, TCGA-Q1-A6DW-01A, TCGA-Q1-A73O-01A, TCGA-Q1-A73P-01A, TCGA-Q1-A73Q-01A, TCGA-Q1-A73R-01A, TCGA-Q1-A73S-01A, TCGA-R2-A69V-01A, TCGA-RA-A741-01A, TCGA-UC-A7PD-01A, TCGA-UC-A7PF-01A, TCGA-UC-A7PG-01A, TCGA-UC-A7PI-01A, TCGA-VS-A8EB-01A, TCGA-VS-A8EC-01A, TCGA-VS-A8EG-01A, TCGA-VS-A8EH-01A, TCGA-VS-A8EI-01A, TCGA-VS-A8EK-01A, TCGA-VS-A8EL-01A, TCGA-VS-A8Q8-01A, TCGA-VS-A8Q9-01A, TCGA-VS-A8QA-01A, TCGA-VS-A8QC-01A, TCGA-VS-A8QF-01A, TCGA-VS-A8QM-01A, TCGA-VS-A94W-01A, TCGA-VS-A94X-01A, TCGA-VS-A94Y-01A, TCGA-VS-A94Z-01A, TCGA-VS-A950-01A, TCGA-VS-A952-01A, TCGA-VS-A953-01A, TCGA-VS-A954-01A, TCGA-VS-A957-01A, TCGA-VS-A958-01A, TCGA-VS-A959-01A, TCGA-VS-A9U5-01A, TCGA-VS-A9U6-01A, TCGA-VS-A9U7-01A, TCGA-VS-A9UB-01A, TCGA-VS-A9UC-01A, TCGA-VS-A9UD-01A, TCGA-VS-A9UH-01A, TCGA-VS-A9UI-01A, TCGA-VS-A9UL-01A, TCGA-VS-A9UM-01A, TCGA-VS-A9UO-01A, TCGA-VS-A9UP-01A, TCGA-VS-A9UQ-01A, TCGA-VS-A9UR-01A, TCGA-VS-A9UU-01A, TCGA-VS-A9UV-01A, TCGA-VS-A9UY-01A, TCGA-VS-A9UZ-01A, TCGA-VS-A9V1-01A, TCGA-VS-A9V2-01A, TCGA-VS-A9V3-01A, TCGA-VS-A9V5-01A, TCGA-VS-AA62-01A, TCGA-WL-A834-01A, TCGA-XS-A8TJ-01A, TCGA-ZJ-A8QO-01A, TCGA-ZJ-A8QQ-01A, TCGA-ZJ-A8QR-01A, TCGA-ZJ-AAX4-01A, TCGA-ZJ-AAXA-01A, TCGA-ZJ-AAXB-01A, TCGA-ZJ-AAXD-01A, TCGA-ZJ-AAXF-01A, TCGA-ZJ-AAXI-01A, TCGA-ZJ-AAXJ-01A, TCGA-ZJ-AAXN-01A, TCGA-ZJ-AAXT-01A, TCGA-ZJ-AAXU-01A, TCGA-ZJ-AB0H-01A, TCGA-ZJ-AB0I-01A, TCGA-ZX-AA5X-01A |
| negative | 22 | TCGA-2W-A8YY-01A, TCGA-C5-A2LT-01A, TCGA-C5-A8YQ-01A, TCGA-DS-A3LQ-01A, TCGA-EA-A410-01A, TCGA-EA-A4BA-01A, TCGA-EA-A556-01A, TCGA-EK-A2PM-01A, TCGA-FU-A3HZ-01A, TCGA-FU-A57G-01A, TCGA-HM-A6W2-01A, TCGA-IR-A3LA-01A, TCGA-JW-A5VH-01A, TCGA-JW-A5VI-01A, TCGA-JW-A5VK-01A, TCGA-VS-A8EJ-01A, TCGA-VS-A8QH-01A, TCGA-VS-A9UJ-01A, TCGA-VS-A9UT-01A, TCGA-VS-A9V0-01A, TCGA-VS-A9V4-01A, TCGA-ZJ-AAX8-01A |

**Supplementary Table 3:**

The GO enrichmen analysis of biological process based on genes with higher levels of m6A methylation in SiHa-lv-shFTO.

| GO.ID^1^ | Term^2^ | Count^3^ | Fold.Enrichment^4^ | Pvalue^5^ | FDR^6^ | Enrichment.Score^7^ | Gene.Ratio^8^ |
| --- | --- | --- | --- | --- | --- | --- | --- |
| GO:0032501 | multicellular organismal process | 130 | 1.141435435 | 0.028545459 | 0.562770647 | 1.544462964 | 0.460992908 |
| GO:0044707 | single-multicellular organism process | 126 | 1.145737573 | 0.02854559 | 0.562770647 | 1.54446097 | 0.446808511 |
| GO:0019222 | regulation of metabolic process | 125 | 1.185883288 | 0.009353877 | 0.432894243 | 2.029008359 | 0.443262411 |
| GO:0051716 | cellular response to stimulus | 125 | 1.126607822 | 0.048502937 | 0.687985817 | 1.314231962 | 0.443262411 |
| GO:0032502 | developmental process | 122 | 1.350923689 | 4.30974E-05 | 0.040482014 | 4.365548731 | 0.432624113 |
| GO:0044767 | single-organism developmental process | 121 | 1.353485031 | 4.36088E-05 | 0.040482014 | 4.360426274 | 0.429078014 |
| GO:0080090 | regulation of primary metabolic process | 118 | 1.241227418 | 0.002469623 | 0.238257049 | 2.607369401 | 0.418439716 |
| GO:0031323 | regulation of cellular metabolic process | 116 | 1.226963479 | 0.004244429 | 0.289534417 | 2.372180765 | 0.411347518 |
| GO:0007275 | multicellular organismal development | 110 | 1.410081451 | 2.08365E-05 | 0.040482014 | 4.681175119 | 0.390070922 |
| GO:0060255 | regulation of macromolecule metabolic process | 110 | 1.242108493 | 0.003810107 | 0.277426561 | 2.419062875 | 0.390070922 |
| GO:0048856 | anatomical structure development | 102 | 1.276889311 | 0.002369933 | 0.238257049 | 2.625263992 | 0.361702128 |
| GO:0048731 | system development | 95 | 1.395569153 | 0.000167406 | 0.083191043 | 3.776228985 | 0.336879433 |
| GO:0048518 | positive regulation of biological process | 92 | 1.24465819 | 0.009178346 | 0.428401262 | 2.037235573 | 0.326241135 |
| GO:0019219 | regulation of nucleobase-containing compound metabolic process | 91 | 1.244383081 | 0.009700343 | 0.44354447 | 2.013212915 | 0.322695035 |
| GO:0051171 | regulation of nitrogen compound metabolic process | 91 | 1.217014805 | 0.017525504 | 0.514057128 | 1.756329475 | 0.322695035 |
| GO:0034645 | cellular macromolecule biosynthetic process | 91 | 1.166521928 | 0.04829192 | 0.687985817 | 1.316125523 | 0.322695035 |
| GO:0048522 | positive regulation of cellular process | 86 | 1.303905412 | 0.003428806 | 0.271263696 | 2.464857032 | 0.304964539 |
| GO:0030154 | cell differentiation | 83 | 1.451283169 | 0.000144259 | 0.083191043 | 3.840857116 | 0.294326241 |
| GO:0048869 | cellular developmental process | 83 | 1.367217487 | 0.001028448 | 0.174618315 | 2.987817705 | 0.294326241 |
| GO:0010556 | regulation of macromolecule biosynthetic process | 80 | 1.237220227 | 0.018667926 | 0.528215259 | 1.728903924 | 0.283687943 |
| GO:2000112 | regulation of cellular macromolecule biosynthetic process | 79 | 1.259934817 | 0.012718306 | 0.466383847 | 1.895570737 | 0.280141844 |
| GO:0051252 | regulation of RNA metabolic process | 77 | 1.290321675 | 0.007894378 | 0.406709399 | 2.102682103 | 0.273049645 |
| GO:0032774 | RNA biosynthetic process | 76 | 1.231328702 | 0.02460639 | 0.542290231 | 1.608952104 | 0.269503546 |
| GO:0006351 | transcription, DNA-templated | 75 | 1.255702427 | 0.016694382 | 0.514057128 | 1.777429643 | 0.265957447 |
| GO:0006796 | phosphate-containing compound metabolic process | 75 | 1.207256571 | 0.038529474 | 0.64613462 | 1.414206915 | 0.265957447 |
| GO:0006793 | phosphorus metabolic process | 75 | 1.192480691 | 0.0490561 | 0.692236074 | 1.309306984 | 0.265957447 |
| GO:0006355 | regulation of transcription, DNA-templated | 74 | 1.286829483 | 0.009990106 | 0.44354447 | 2.000429897 | 0.262411348 |
| GO:2001141 | regulation of RNA biosynthetic process | 74 | 1.271358134 | 0.01323741 | 0.466383847 | 1.878196978 | 0.262411348 |
| GO:0048513 | organ development | 66 | 1.318606052 | 0.009147678 | 0.428401262 | 2.038689131 | 0.234042553 |
| GO:0010646 | regulation of cell communication | 65 | 1.407871292 | 0.002117894 | 0.238257049 | 2.674095819 | 0.230496454 |
| GO:0023051 | regulation of signaling | 64 | 1.389370591 | 0.003179059 | 0.259117013 | 2.497701445 | 0.226950355 |
| GO:0009653 | anatomical structure morphogenesis | 61 | 1.503550687 | 0.000595808 | 0.135611241 | 3.224893735 | 0.216312057 |
| GO:0009966 | regulation of signal transduction | 59 | 1.43384987 | 0.002343416 | 0.238257049 | 2.630150559 | 0.209219858 |
| GO:0065009 | regulation of molecular function | 58 | 1.352613349 | 0.008990674 | 0.428401262 | 2.046207765 | 0.205673759 |
| GO:0009893 | positive regulation of metabolic process | 57 | 1.350226169 | 0.009971639 | 0.44354447 | 2.001233455 | 0.20212766 |
| GO:0010604 | positive regulation of macromolecule metabolic process | 55 | 1.444086631 | 0.002898959 | 0.247362721 | 2.537757952 | 0.195035461 |
| GO:0007399 | nervous system development | 54 | 1.586134132 | 0.000370593 | 0.121379497 | 3.431102309 | 0.191489362 |
| GO:0031325 | positive regulation of cellular metabolic process | 52 | 1.301926357 | 0.026167789 | 0.553344542 | 1.582232977 | 0.184397163 |
| GO:0048468 | cell development | 50 | 1.586884355 | 0.000628218 | 0.137227937 | 3.201889633 | 0.177304965 |

^1^: GO.ID stands for the ID of gene ontology term.

^2^: Term stands for the name of gene ontology term.

^3^: Count stands for the number of genes associated with the listed GO.ID.

^4^: Fold.Enrichment stands for the Fold Enrichment value of the GOID, and it equals (Count/Pop.Hits)/(List.Total/Pop.Total).

^5^: Pvalue stands for the significance testing value of the GOID.

^6^: FDR stands for the false discovery rate of the GOID, using Benjamini & Hochberg (1995) method.

^7^: Enrichment.Score stands for the Enrichment Score value of the GOID, it equals (-log10(Pvalue)). Gene.

^8^: Ratio stands for the Gene Ratio value genes associated with the GOID, it equals (Count/List.Total).

**Supplementary Table 4:**

The KEGG enrichmen analysis based on genes with higher levels of m6A methylation in SiHa-lv-shFTO.

| PathwayID^1^ | Definition^2^ | Fisher-  Pvalue^3^ | Selection  Counts^4^ | Selection  Size^5^ | Count^6^ | Size^7^ | FDR^8^ | Enrichment_  Score^9^ | GeneRatio^10^ |
| --- | --- | --- | --- | --- | --- | --- | --- | --- | --- |
| hsa05162 | Measles | 0.006011773 | 7 | 112 | 136 | 6990 | 0.6853339 | 2.220997 | 0.0625 |
| hsa04930 | Type II diabetes mellitus | 0.00706828 | 4 | 112 | 48 | 6990 | 0.6853339 | 2.150686 | 0.035714 |
| hsa04261 | Adrenergic signaling in cardiomyocytes | 0.009415991 | 7 | 112 | 148 | 6990 | 0.6853339 | 2.026134 | 0.0625 |
| hsa04925 | Aldosterone synthesis and secretion | 0.009419053 | 5 | 112 | 81 | 6990 | 0.6853339 | 2.025993 | 0.044643 |
| hsa04024 | cAMP signaling pathway | 0.01425354 | 8 | 112 | 199 | 6990 | 0.6853339 | 1.846077 | 0.071429 |
| hsa05205 | Proteoglycans in cancer | 0.01590577 | 8 | 112 | 203 | 6990 | 0.6853339 | 1.798445 | 0.071429 |
| hsa05160 | Hepatitis C | 0.01961875 | 6 | 112 | 133 | 6990 | 0.6853339 | 1.707329 | 0.053571 |
| hsa04068 | FoxO signaling pathway | 0.02027825 | 6 | 112 | 134 | 6990 | 0.6853339 | 1.69297 | 0.053571 |
| hsa05164 | Influenza A | 0.02193294 | 7 | 112 | 175 | 6990 | 0.6853339 | 1.658903 | 0.0625 |
| hsa04066 | HIF-1 signaling pathway | 0.02443863 | 5 | 112 | 103 | 6990 | 0.6853339 | 1.611923 | 0.044643 |
| hsa05202 | Transcriptional misregulation in cancer | 0.02512891 | 7 | 112 | 180 | 6990 | 0.6853339 | 1.599826 | 0.0625 |
| hsa04931 | Insulin resistance | 0.03026803 | 5 | 112 | 109 | 6990 | 0.7225168 | 1.519016 | 0.044643 |
| hsa04725 | Cholinergic synapse | 0.03239012 | 5 | 112 | 111 | 6990 | 0.7225168 | 1.489588 | 0.044643 |
| hsa04390 | Hippo signaling pathway | 0.03678171 | 6 | 112 | 154 | 6990 | 0.7225168 | 1.434368 | 0.053571 |
| hsa04921 | Oxytocin signaling pathway | 0.0408839 | 6 | 112 | 158 | 6990 | 0.7225168 | 1.388448 | 0.053571 |
| hsa04071 | Sphingolipid signaling pathway | 0.04307849 | 5 | 112 | 120 | 6990 | 0.7225168 | 1.36574 | 0.044643 |
| hsa05410 | Hypertrophic cardiomyopathy (HCM) | 0.04371348 | 4 | 112 | 83 | 6990 | 0.7225168 | 1.359385 | 0.035714 |
| hsa04350 | TGF-beta signaling pathway | 0.04535267 | 4 | 112 | 84 | 6990 | 0.7225168 | 1.343397 | 0.035714 |

^1^. PathwayID stands for Pathway identifiers used in KEGG.

^2^. Definition stands for the definition of the PathwayID.

^3^. Fisher-Pvalue stands for the enrichment p-value of the PathwayID used Fisher's exact test.

^4^. SelectionCounts stands for the Count of the DE genes' entities directly associated with the listed PathwayID.

^5^. SelectionSize stands for the total number of the DE genes' entities.

^6^. Count stands for the count of the chosen background population genes' entities associated with the listed PathwayID.

^7^. Size stands for the total number of chosen background population genes' entities.

^8^. FDR stands for the false discovery rate of the PathwayID.

^9^. Enrichment_Score stands for the Enrichment Score value of the PathwayID, and it equals (-log10(Pvalue)).

^10^. GeneRatio stands for Gene Ratio value of the PathwayID, and it equals (SelectionCounts/SelectionSize).
